# Longer prime presentation decreases picture–word cross-domain priming

**DOI:** 10.3389/fpsyg.2015.01040

**Published:** 2015-07-21

**Authors:** Kiyofumi Miyoshi, Yusuke Kimura, Hiroshi Ashida

**Affiliations:** Laboratory of Psychology, Department of Letters, Kyoto University, Kyoto, Japan

**Keywords:** adaptation, habituation, priming, implicit memory, reaction time

## Abstract

A short prime presentation has been shown to provide a greater priming magnitude, whereas a longer prime presentation results in a lower priming magnitude. In Experiment 1, we attempted to replicate the decrease of priming using word stimuli. Words were presented in both prime and test sessions, and participants judged whether each stimulus was natural or manmade. In Experiment 2, we employed a cross-domain priming paradigm to assess the impact of prime duration on non-perceptual processes. Pictures were presented in prime sessions, and their semantically matched words were presented in test sessions. We did not observe a significant decrease in priming in Experiment 1. However, we found that 2000 ms of prime exposure led to weaker cross-domain priming when compared with 250 ms of the exposure in Experiment 2. The results suggest that the longer presentation of pictures causes a non-perceptual adaptation effect. This effect may occur at conceptual, linguistic, and/or response-related levels.

## Introduction

Priming refers to improved processing of a repeated stimulus [e.g., greater accuracy or faster reaction time (RT)] through unconscious memory retrieval (see Schacter et al., 2007, for a review). Many studies suggest that presenting stimuli for a short duration provides positive priming, whereas presenting stimuli for a long duration provides lower or even negative priming (Huber and O’Reilly, 2003; Zago et al., 2005; Voss and Gonsalves, 2010; Faivre and Kouider, 2011; Miyoshi and Ashida, 2014). Huber and O’Reilly (2003) proposed a neural network model of perceptual identification to explain the impact of prime duration on short-term priming. In this model, short prime presentation leads to the pre-activation of stimulus representations and provides a “head start” for stimulus processing, resulting in a positive priming effect. However, longer prime presentation leads to the adaptation of stimulus representations and reduces the magnitude of pre-activation, resulting in a lower or negative priming effect. In this model, pre-activation and adaptation are considered to be two different mechanisms which can simultaneously coexist. Note that considering the evidence that short- and long-term priming were associated with different neural repetition suppression effects (Epstein et al., 2008), this model may be only applicable to data from short-term priming experiments.

Zago et al. (2005) investigated the impact of prime duration on long-term repetition priming in a natural versus manmade decision task for pictures of familiar objects. Their results demonstrated that the magnitudes of behavioral priming were lower for longer presentation of primes (350–1900 ms) than for shorter presentations (250 ms). Consistent with the behavioral results, the magnitude of functional magnetic resonance imaging (fMRI) repetition suppression effects was lower for longer prime presentations than shorter presentations. It should be noted that these researchers conducted three different versions of the experiment, each of which has different inter-repetition intervals between the prime and target: average time between presentations for the block design fMRI experiment was 40–58 s (9–18 intervening stimuli); for the event-related fMRI experiment was 2 s–14 min; (1–374 intervening stimuli); and for the behavioral experiment was 2 s–2 min (2–60 intervening stimuli). Notably, these different versions of the experiment yielded highly similar results, suggesting the robustness of results.

Although the focus of their study was to investigate how visual exposure shapes perceptual representations of objects, the researchers found fMRI repetition suppression effects in cortical regions typically associated with perceptual processes (posterior occipital–temporal regions) and non-perceptual (e.g., conceptual, linguistic, and response-related) processes (anterior temporal and inferior frontal regions). Thus, the need for additional research to investigate the extent to which the priming decrease with longer prime presentation reflects general operating characteristics in different levels of processing was emphasized. For that purpose, behavioral evidence about the impact of prime duration on high level priming is needed, because fMRI repetition suppression and behavioral priming are not always associated (Sayres and Grill-Spector, 2006).

The precise mechanisms for the “rise and fall” pattern of long-term priming are still unclear (see Zago et al., 2005; Voss and Gonsalves, 2010; Miyoshi and Ashida, 2014). However, we hypothesized that bottom-up changes in stimulus processing are responsible for the pattern (Miyoshi and Ashida, 2014); short stimulus presentation times lead to the fine tuning of the neural activities coding the stimulus (sharpening; Desimone, 1996; Wiggs and Martin, 1998), but longer stimulus presentation times lead to the fatigue of neurons (adaptation) and make stimulus representations less efficient.

In the present study, we conducted two experiments. In Experiment 1, we attempted to assess the generality of the rise and fall of long-term priming reported in Zago et al. (2005). Specifically, we attempted to replicate the decrease of long-term priming with longer prime duration using word stimuli. Words were presented in both prime and test sessions, and participants were required to identify them as natural or manmade. Following the method of Voss and Gonsalves (2010), the prime duration was set at 250 and 2000 ms. In Experiment 2, we employed a long-term cross-domain priming paradigm to investigate the effect of stimulus duration on high-level processes beyond the perceptual level. Stimuli comprised pictures of real-world objects and semantically matched words. Pictures were presented in prime sessions, but the semantically matched words were presented in test sessions, which should exclude perceptual priming/adaptation and involve only non-perceptual (e.g., conceptual, linguistic, and response-related) priming/adaptation (see Schacter et al., 2004, for a review of different types of priming). This cross-domain paradigm serves the purpose of investigating the temporal dynamics of high-level stimulus representations.

## Materials and Methods

### Participants and Design

Sixteen undergraduates (eight men, eight women; ages 19–24 years) volunteered to participate in both Experiments. The sample size was determined according to above mentioned previous studies (12–20 in Zago et al., 2005; 14 in Voss and Gonsalves, 2010). The order of Experiments 1 and 2 was counterbalanced across the participants. The participants received 1000 yen for their participation. All participants had normal color vision; an informed written consent was obtained from them before the experiments. All data were collected in accordance with the ethical principles of the Japanese Psychological Association. The study was a 2 (experiment: Experiments 1 and 2) × 3 (prime condition: novel, 250 ms, and 2000 ms) design with both variables as within-participant factors.

### Materials and Procedure

We used 300 color pictures of real-world objects and 300 of their semantically matched Japanese words. We organized two stimulus lists, each including 150 semantically matched pairs of a picture and a word. Each list included an equal number of natural and manmade pairs, and the number of letters of the word stimuli was counterbalanced between the lists. The lists were counterbalanced such that they were used in Experiments 1 and 2 for an equal number of times across the participants. The stimulus pairs in each list were randomly assigned to each prime condition (novel, 250 ms, and 2000 ms) for each participant. The stimuli were displayed on a dark background on a computer monitor using the software Presentation (Neurobehavioral Systems). The distance between the monitor and participant was 48 cm. Each picture was framed in a white window measuring 7.5 (width) cm × 7.5 (height) cm (8.9° × 8.9° of visual angle). Each word measured 2.2–5.5 × 1.1 cm (2.6–6.6° × 1.3° of visual angle).

The participants took part in both Experiments. Each experiment contained two consecutive sets of a prime–test session. Each prime session included 50 trials, and each test session included 75 trials. Thus, the participants experienced a total of 250 trials in each experiment. The order of Experiments 1 and 2 was counterbalanced across the participants, and there was a 3-min interval between the two experiments.

In Experiment 1, 50 words were presented one by one; this was immediately followed by a mask in the prime session. The participants were required to answer as quickly and accurately as possible whether each word was natural or manmade with a button press. Half the words were presented for 250 ms, and the remaining were presented for 2000 ms. The duration of the mask was either 2250 or 500 ms, thereby ensuring that the total duration of each trial was 2500 ms. Trial presentation order was randomized, and intertrial intervals were 2000 ms.

The test session began 15 s after the prime session. In each test session, 25 novel words, 25 250-ms-primed words, and 25 2000-ms-primed words were presented one by one for 500 ms in a random order; this was immediately followed by the mask. The duration of the mask was 1000 ms. The participants were again required to answer as quickly and accurately as possible whether each word was natural or manmade with a button press; intertrial intervals were 2000 ms.

The procedure of Experiment 2 was the same as that of Experiment 1, except that pictures were presented in the prime sessions and their semantically matched words were presented in the test sessions.

## Results

**FIGURE 1:**
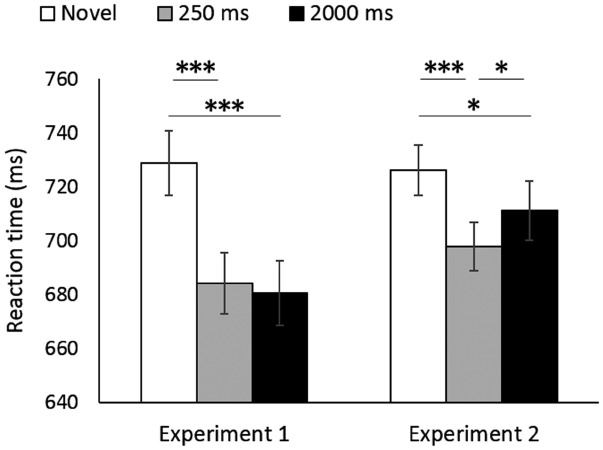
**Mean reaction time in each condition.** Error bars indicate the 95% within-participant confidence interval (Baguley, 2012). ***p < 0.001, *p < 0.05.

Table 1 presents mean response accuracies recorded in the test sessions. RT analyses included only trials with correct responses. We conducted a repeated-measures ANOVA on the mean RTs with experiment (Experiments 1 and 2) and prime condition (novel, 250 ms, and 2000 ms) as within-participant factors (Figure 1). There was no significant main effect of experiment [*F*(1,15) = 1.98, *p* = 0.18, ${\text{η}}_{p}^{2}$ = 0.117], but there was a significant main effect of prime condition [*F*(2,30) = 27.70, *p* < 0.001, ${\text{η}}_{p}^{2}$ = 0.649]. The interaction between these two factors was significant [*F*(2,30) = 5.13, *p* = 0.01, ${\text{η}}_{p}^{2}$ = 0.255].

**Table 1:** **Mean response accuracy in each condition**.

	**Novel**	**250 ms**	**2000 ms**
Experiment 1	0.93 (0.14)	0.94 (0.09)	0.94 (0.11)
Experiment 2	0.95 (0.07)	0.96 (0.06)	0.95 (0.05)

Standard deviation (SD) is shown in parentheses.

There was a significant simple main effect of prime condition in Experiment 1 [*F*(2,30) = 18.63, *p* < 0.001, ${\text{η}}_{p}^{2}$ = 0.554]. Paired *t*-tests with Shaffer’s modified sequentially rejective Bonferroni procedure (Shaffer, 1986) revealed that the mean RT in the novel condition was significantly longer than those in the 250 ms condition [*t*(15) = 5.93, *p* < 0.001, *d′* = 1.48] and the 2000 ms condition [*t*(15) = 5.85, *p* < 0.001, *d′* = 1.46]. There was no significant difference in the mean RTs of the 250 and 2000 ms conditions [*t*(15) = 0.35, *p* = 0.73, *d′* = 0.09]. The results revealed that the longer presentation of word primes did not decrease the priming magnitude in Experiment 1.

A simple main effect of prime condition was also significant in Experiment 2 [*F*(2,30) = 12.09, *p* < 0.001, ${\text{η}}_{p}^{2}$ = 0.446]. Paired *t*-tests with Shaffer’s modified sequentially rejective Bonferroni procedure revealed that the mean RT in the novel condition was significantly longer than those in the 250 ms condition [*t*(15) = 5.27, *p* < 0.001, *d′* = 1.32] and the 2000 ms condition [*t*(15) = 2.30, *p* = 0.04, *d′* = 0.58]. Notably, the mean RT in the 2000 ms condition was significantly longer than that in the 250 ms condition [*t*(15) = 2.50, *p* = 0.02, *d′* = 0.63], thereby suggesting that the longer presentation of picture primes decreased the magnitude of long-term cross-domain priming.

Finally, we conducted a repeated-measures ANOVA on the mean response accuracies with experiment and prime condition as within-participant factors. There was no significant main effect of experiment [*F*(1,15) = 1.06, *p* = 0.32, ${\text{η}}_{p}^{2}$ = 0.066] and prime condition [*F*(2,30) = 0.35, *p* = 0.71, ${\text{η}}_{p}^{2}$ = 0.023]. The interaction between these two factors was not significant [*F*(2,30) = 0.03, *p* = 0.97, ${\text{η}}_{p}^{2}$ = 0.002]. The results suggest that RT differences cannot be attributed to a speed versus accuracy trade-off.

## Discussion

In Experiment 1, mean RTs were highly matched between the 250 and 2000 ms conditions. Namely, we did not observe the decrease of long-term word repetition priming with longer prime duration. Contrary to the present results, Zago et al. (2005) demonstrated that longer prime presentation decreases the magnitude of long-term picture repetition priming. These different result patterns probably arise because the processing of words and pictures depends on (at least partially) different underlying mechanisms. However, it is unsafe to further infer about the difference between our study and Zago et al. (2005) on the basis of the negative results reported here. Considering the relatively small sample size of the present study, the non-significant RT difference between the 250 and 2000 ms conditions could simply result from a lack of statistical power. The power to detect this RT difference is 0.65, if we set the significance level at 0.05 and the population effect size (*δ*′) at 0.63 by reference to the results of Experiment 2.

To the best of our knowledge, Experiment 2 provides the first behavioral evidence that the longer presentation of picture primes decreases the magnitude of picture–word cross-domain priming. The present behavioral results are consistent with the fMRI results of Zago et al. (2005) that longer presentations of picture primes decrease the magnitude of neural repetition suppression in cortical regions typically associated with non-perceptual processes (anterior temporal and inferior frontal regions). Taken together, these results provide strong evidence about the temporal dynamics of high-level stimulus representations. As mentioned in the introduction, we hypothesized that bottom-up changes in stimulus processing are responsible for the decrease of long-term priming with longer prime duration (Miyoshi and Ashida, 2014). According to the hypothesis, the present results suggest that short presentation of pictures leads to the fine tuning of the neural activities which code higher-level information beyond the perceptual level; however, longer presentation times lead to adaptation at higher-levels, such as for conceptual, linguistic, and response-related processes. The combination of these two may be responsible for the long-lasting changes in stimulus representations.

Alternatively, Miyoshi and Ashida (2014) suggested that another possible cause of the priming decrease is interference from explicit memory retrieval. We hypothesized that longer prime presentation enables an elaborate explicit memory encoding and that explicit recollection during the test might interfere with implicit memory processes and decrease the magnitude of priming. In fact, explicit recollection is considered to be dependent on slow and effortful processes (Hasher and Zacks, 1979; Jacoby, 1991). From this perspective, this study’s results suggest that explicit memory retrieval interferes with non-perceptual implicit memory.

The present study provides the novel finding that the longer presentation of pictures reduces the magnitude of long-term cross-domain priming, thereby suggesting the effect of high-level adaptation. However, the present findings do not identify the exact level of processing at which the present adaptation effect occurs. Future research is required to further elucidate this point.

### Conflict of Interest Statement

The authors declare that the research was conducted in the absence of any commercial or financial relationships that could be construed as a potential conflict of interest.
